# Lipotoxic Effect of p21 on Free Fatty Acid-Induced Steatosis in L02 Cells

**DOI:** 10.1371/journal.pone.0096124

**Published:** 2014-04-30

**Authors:** Jie-wei Wang, Xing-yong Wan, Hua-tuo Zhu, Chao Lu, Wei-lai Yu, Chao-hui Yu, Zhe Shen, You-ming Li

**Affiliations:** Department of Gastroenterology, First Affiliated Hospital, College of Medicine, Zhejiang University, Hangzhou, China; Case Western Reserve University, United States of America

## Abstract

Nonalcoholic fatty liver disease (NAFLD) is increasingly regarded as a hepatic manifestation of metabolic syndrome. Though with high prevalence, the mechanism is poorly understood. This study aimed to investigate the effects of p21 on free fatty acid (FFA)-induced steatosis in L02 cells. We therefore analyzed the L02 cells with MG132 and siRNA treatment for different expression of p21 related to lipid accumulation and lipotoxicity. Cellular total lipid was stained by Oil Red O, while triglyceride content, cytotoxicity assays, lipid peroxidation markers and anti-oxidation levels were measured by enzymatic kits. Treatment with 1 mM FFA for 48 hr induced magnificent intracellular lipid accumulation and increased oxidative stress in p21 overload L02 cells compared to that in p21 knockdown L02 cells. By increasing oxidative stress and peroxidation, p21 accelerates FFA-induced lipotoxic effect in L02 cells and might provide information about potentially new targets for drug development and treatments of NAFLD.

## Introduction

Nonalcoholic fatty liver disease (NAFLD) represents a spectrum of liver damage in individuals encompassing fatty liver (simple steatosis), steatohepatits (NASH) and cirrhosis in the absence of alcohol abuse [1]. NAFLD is an emerging health problem worldwide, estimated prevalence ranges from 6.3% to 33% with a median of 20% in the general population [2]. Population studies show that NAFLD is strongly associated with obesity, dyslipidemia, insulin resistance, and hypertension. Indeed, NAFLD is thought of as the hepatic manifestation of the metabolic syndrome[3]. The exact mechanisms underlying NAFLD and treatment options are so far poorly understood. The pathogenesis may be explained by the ‘two-hit’ hypothesis, where the ‘first hit’ consists in inflammation and development of steatosis, which sensitizes the liver to a variety of ‘second hits’ lead to fibrosis[4].

These ‘two hits’ consist of the excessive hepatic fat accumulation owing to insulin resistance and oxidative stress [5]. Oxidative stress can result from either excess reactive oxygen species (ROS) production and/or deficient antioxidant capacity [6]. A growing body of evidence now strongly suggests that oxidative stress-induced lipid peroxidation (LPO) plays a role in the liver injury occurring in NAFLD. Several different animal models of NAFLD have provided persuasive evidence that ROS-mediated lipid peroxidation occurs [7], [8]. It is well accepted that NAFLD patients exhibit an elevated lipolysis and high circulating free fatty acid (FFA) levels [9]. Sanyal et al have suggested that increased mitochondrial β-oxidation of FFA may be an important source of ROS in NASH [10]. This is consistent with an in vitro study demonstrating that incubating hepatocytes with FFA increases their ROS formation [11].

p21^Waf1/Cip1/Sdi1^ is a well-characterized cyclin-dependent kinase inhibitor (CKI) that belongs to the Cip/Kip family of cdk inhibitors [12]. The p21 (CIP1/WAF1) protein negatively modulates cell cycle progression at S phase and blocks DNA synthesis [13]. In addition to growth arrest, p21 is also important in the stress response [14] and is a transcriptional target of the tumor suppressor gene, p53 [15].

Recent studies have connected p21 with NAFLD. In ob/ob mice, NAFLD is associated with increased expression of p21, which together with STAT-3 overexpression impaired regeneration of fatty livers [16]. In rats, p21^Cip1^ mRNA levels were significantly higher in livers of OP (obesity prone) models compared with OR (obesity-resistant) models [17]. In human NAFLD liver samples, expression levels of p21 were significantly elevated compared with results obtained from normal liver samples [18]. And in patients with NASH, hepatic expression level of p21 is significantly higher than patients with simple steatosis [18], [19] and impaired hepatocyte replication by p21 expression correlates with insulin resistance and inflammatory activity [19]. Aravinthan's study demonstrated that markers of senescence including hepatocyte p21 expression predicted an adverse liver-related outcome in patients with NAFLD [20]. Moreover, it is confirmed that p21 is necessary for the induction of ROS and mitochondrial dysfunction observed in senescence [21]. It has also been postulated that p21 induced oxidative stress may be responsible for impaired hepatocyte regeneration [22], [23].

These findings suggest a possible link between p21 activity, ROS generation, LPO induction, and FFA-induced liver injury. The aim of this study was to investigate the role of p21 in the establishment of hepatocyte steatosis initiated by ROS, LPO and analyze the possible way.

Cellular FFA loading was utilized because it appears to reproduce several of the key features of hepatic steatosis in human beings [24]. Post-transcriptional control of p21 has two ways, ubiquitin-dependent and ubiquitin-independent proteolysis. We choose N-benzyloxycarbony (Cbz)-Leu-Leu-Leucinal (MG132) as the ubiquitin-proteasome pathway inhibitor to testify whether overload of p21 activity could modulate ROS-dependent toxicity. Accordingly, siRNA transfection was used to explore whether p21 knockdown could correct the steatosis. We found that p21 had a lipotoxic effect on L02 cells buffered in FFA, mainly by increasing oxidative stress and lipid peroxidation.

## Materials and Methods

### A. Culture and treatment of cells

The human non-tumor cell line, L02, was obtained from China Cell Culture Center (Shanghai). The cell was maintained in an atmosphere of 5% CO2 at 37°C in DMEM (high glucose, Gibco) supplemented with 10% fetal bovine serum (FBS; Gibco).

Fat overloading induction of cells was done mainly according to previously established methods [25]. In Brief, L02 cells were exposed to a mixture of FFA (oleate and palmitate, final ration 2∶1; Sigma) at a final concentration of 1 mM for 48 hours.

MG132 is a C-terminal peptide aldehyde and a proteasome substrate analog [26]. It is cell-permeable and blocks proteasome function, without affecting normal biology [27]. For FFA plus MG132 treatments, cells bathing in FFA were incubated in the presence of MG132 (3 µM, Merck) for 48 hours.

### B. siRNA transfection

L02 cells were seeded at 7×10^4^ cells per well into 6-well plates and allowed to adhere by culturing at 37°C, 5% CO2 for 4 hr. p21 siRNA and negative control siRNA were purchased as validated siRNAs (GenePharma). Transient siRNA transfections were carried out using Lipofectamine 2000 (Invitrogen) following the manufacturer's recommendation. The media was changed 12 hr post-transfection, and 24 hr post-transfection, the media was changed with the addition of a final concentration of 1 mM FFA for 48 hr.

### C. Oil Red O staining

Cells were stained with Oil Red O to assess lipid content according to the Sigma protocol. Briefly, cells were washed twice with PBS and fixed with 10% neutral formalin for 15 min, then rinsed with PBS twice again. The fixed cells were stained with freshly prepared Oil Red O solution for 10 min at 37°C. Thereafter, cells were dyed with hematoxylin (JianCheng Bio-technology Institute), followed by washing with PBS before microscopic examination (Nikon microscope).

### D. Measurement of intracellular triglyceride content

Cellular triglyceride (TG) content was measured using an enzymatic kit (Applygen Technologies) following the manufacturer's instructions. Firstly, scraped cells from 6-well tissue culture plats, washed with PBS twice. Afterward, added 100 ul cell lysis buffer and incubated at 70°C for 10 min. Thereafter, centrifuged at 2000 rpm for 5 min, collected the supernatant and measure the TG content by the BCA method (Applygen Technologies).

### E. Cytotoxicity assays

Cell toxicity was routinely measured by assaying aspartate aminotransferase (AST), alanine aminotransferase (ALT; Chemistry analyzer), glutathione (GSH) and oxidized glutathione (GSSG) using kits respectively (A006-2, A061-1, JianCheng Bio-technology Institute), according to the manufacture's recommended protocol.

### F. Lipid peroxidation assay

Production of malondialdehyde (MDA), ROS, LPO and superoxide dismutase (SOD) were assayed using kits respectively (A003-4, E004, A106-1, A006-2, JianCheng Bio-technology Institute), according to the manufacture's recommended protocol.

### G. Quantitative Real-time RT-PCR Analysis

Total RNA from cells was isolated using Trizole reagent (Takara). Random-primed cDNA were generated by reverse transcription of total RNA samples with One Step PrimeScript RT-PCR Kit (Takara). A realtime-PCR analysis was performed with the ABI Prism 7500 sequence Detection System (Applied Biosystems) using SYBR Premix DimerEraser (Takara). GAPDH (for human genes) was used for internal control, respectively. Primers used were as follows: p21, forward 5′-TGTCCGTCAGAACCCATGC-3′, reverse 5′-AAAGTCGAAGTTCCATCGCTC-3′; GAPDH, forward 5′-TCAACGACCACTTTGTCAAGCTCA-3′, reverse 5′- GCTGGTGGTCCAGGGGTCTTACT-3′.

### H. Western blot analysis

After washing cells twice with PBS, whole cell lysates were extracted in M-PER mammalian protein extraction reagent (Thermo Scientific) by centrifugation (12000 rpm, 15 min, 4°C). Cell lysates (20 µg) of total protein were loaded per well and separated on a 10% SDS polyacrylamide gel. Proteins were then transferred to PVDF membrane. Primary antibodies were: anti-p21 antibodies (Epitomics), anti-GAPDH (Cell Signaling Technology). The secondary antibody was a goat anti-rabbit IgG-HRP (Santa Cruz Biotechnology). The membrane was exposed to ECL Hyperfilm (Amersham Biosciences), and the film was developed. Each blot was stripped with a stripping solution (0.1 M glycine, pH 2.9) for 1 hr and re-probed with anti-p21 antibodies to the bands were quantified densitometrically.

## Statistics

Data are expressed as mean ± standard deviation. Statistical analysis was performed using SPSS (version 18). Groups were compared among themselves using the Student's t test. Differences were considered statistically significant at *p* values less than 0.05.

## Results

### A. FFA treatment induces cellular steatosis

**Figure 1:**
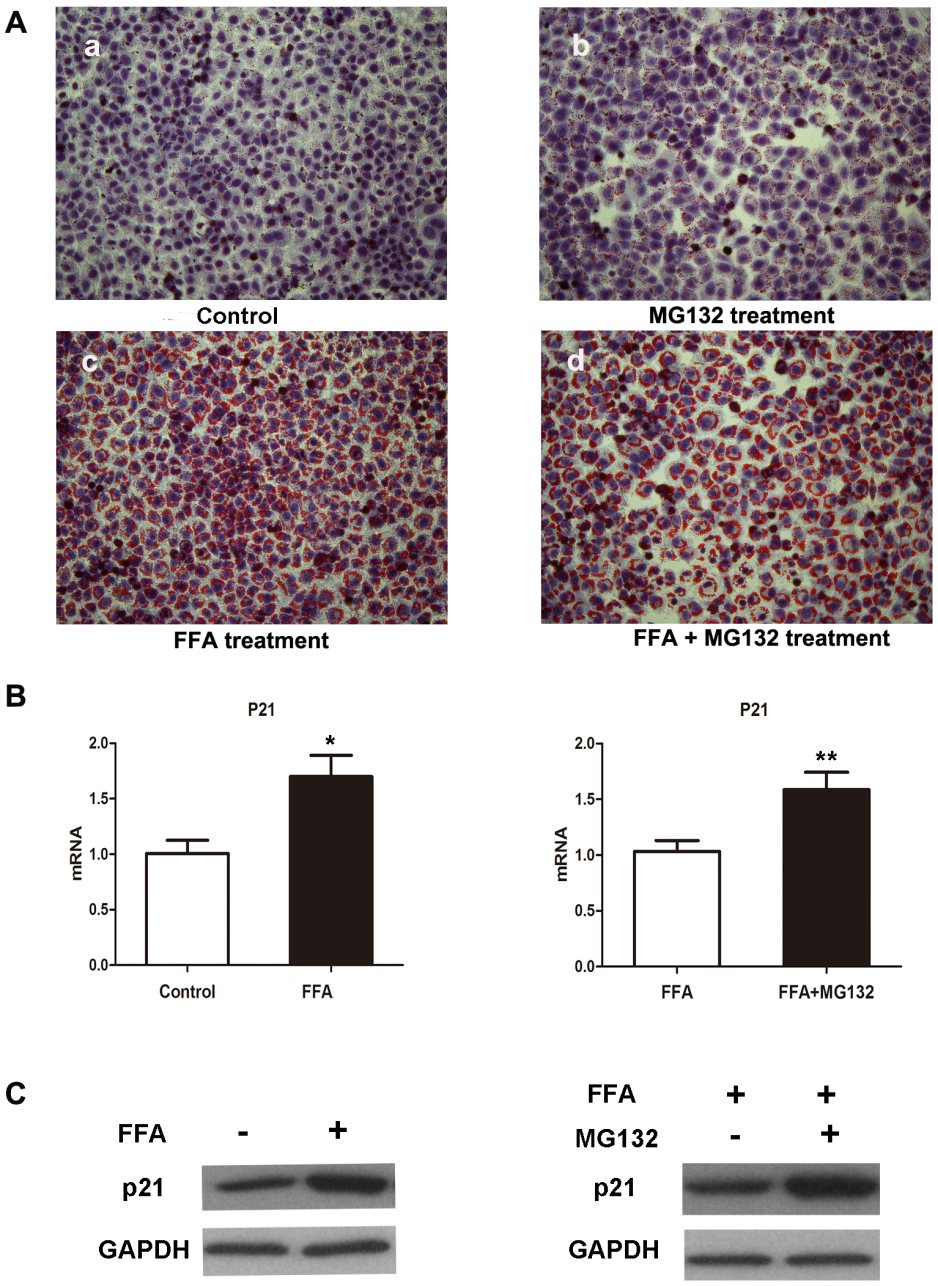
Effects of different treatment on lipid accumulation of L02 cells. (A) Representative micrographs showing intracellular lipid accumulation in L02 cells as stained by Oil Red O (magnification, 200×). Panels a, b, c, d are control cells, cells treated with 3 µM MG132, 1 mM FFA, 1 mM FFA plus 3 µM MG132, respectively for 48 hr. (B) Intracellular mRNA levels of p21 in the indicated groups quantitated by RT-PCR. Values were means ± standard deviation. *p<0.05 compared with L02 cells buffered in DMEM. **p<0.05 compared with L02 cells buffered in FFA. (C) Western blot analysis of cellular p21 protein level in the indicated groups, which were treated with DMEM, 1 mM FFA, 1 mM FFA plus 3 µM MG132 respectively for 48 hr.

It is generally believed that fatty liver results from an imbalance between the hepatic uptake of FFAs, TG synthesis, and excretion [10]. Both in normal people and NAFLD patients, palmitic and oleic acids are the most abundant FFAs in liver triglycerides [28]. According to Gomez Lechon et al's report, hepatocytes loaded with an FFA (1 mM) mixture containing oleate/palmitate (2∶1 ratio) mimics benign chronic steatosis as found in human [25]. Therefore, we established our cell culture model of cellular steatosis by incubating L02 cells with 1 mM FFA (oleate/palmitate, 2∶1). Intracellular lipid vacuoles visible under microscopy, a pathologic hallmark, was confirmed by Oil Red O staining (Figure 1A).

### B. MG132 blocks p21 degradation

As a cell cycle protein, p21 is regulated by proteasomal pathway. MG132 is a peptide aldehyde that inhibits 20S proteasome activity by covalently binding to the active site of the beta subunits and effectively blocks the proteolytic activity of the 26S proteasome complex [15], [29]. The mRNA expression levels of p21 increased in FFA-treated L02 cells, and after MG132 treatment, the p21 mRNA levels were significantly up-regulated (Figure 1B). Western blot analysis also showed that protein levels of p21 were dramatically elevated in L02 cells in response to FFA treatment and turned higher after concurrent treatment with MG132 (Figure 1C). Increased p21 mRNA and protein expression confirmed MG132-induced inhibition of ubiquitin degradation.

### C. p21 overload by MG132 potentiates FFA-induced toxicity in L02 cells

To explore the role of p21 in FFA-treated L02 cells, we compared the cellular lipid accumulation in L02 cells treated with FFA in the presence or absence of MG132 for 48 hours. The results showed that p21 overload increased FFA induced lipid accumulation in L02 cells, which was confirmed by Oil Red O Staining (Figure 1A). Obliviously, there were more intracellular lipid vacuoles with bigger size in MG132 treated L02 cells.

**Figure 2:**
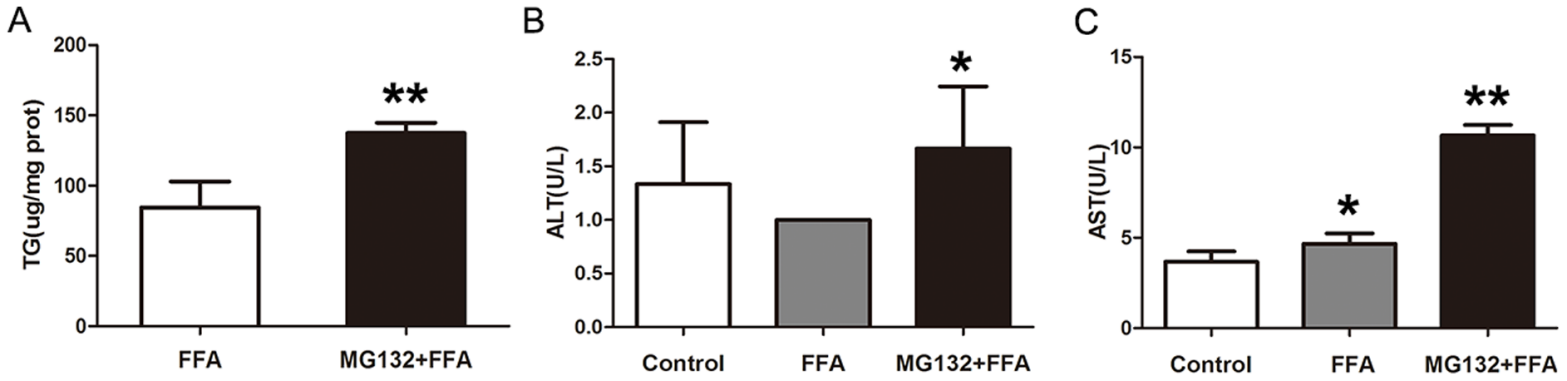
p21 overload potentiates FFA-induced toxicity in L02 cells. (A) TG levels in FFA-buffered L02 cells with or without of MG132 intervene for 48 hr. ALT (B) and AST (C) levels in L02 cells treated with DMEM, 1 mM FFA, 1 mM FFA plus 3 µM MG132 respectively for 48 hr. Results were expressed as means ± standard deviation.*p<0.05 vs DMEM treated L02 cells. **p<0.05 vs FFA treated L02 cells. TG, triglyceride; ALT, alanine aminotransferase; AST, aspartate aminotransferase.

We next measured the intracellular TG levels by an enzymatic kit. The TG content was increased by about 1.63 fold from 84.4 µg/mg protein in FFA-treated cells to 137.5 µg/mg protein in FFA plus MG132-treated cells (Figure 2A). The result presented in Figure 2 showed that AST level significantly increased but not ALT with FFA treatment. But both ALT and AST elevated significantly after FFA plus MG132 intervene. It seems that p21 overload and FFA produce a synergistic toxicity in L02 cells that is greater than that found in control L02 cells or in FFA alone group.

### D. p21 overload aggravates oxidative stress accumulation in FFA-buffered L02 cells

**Figure 3:**
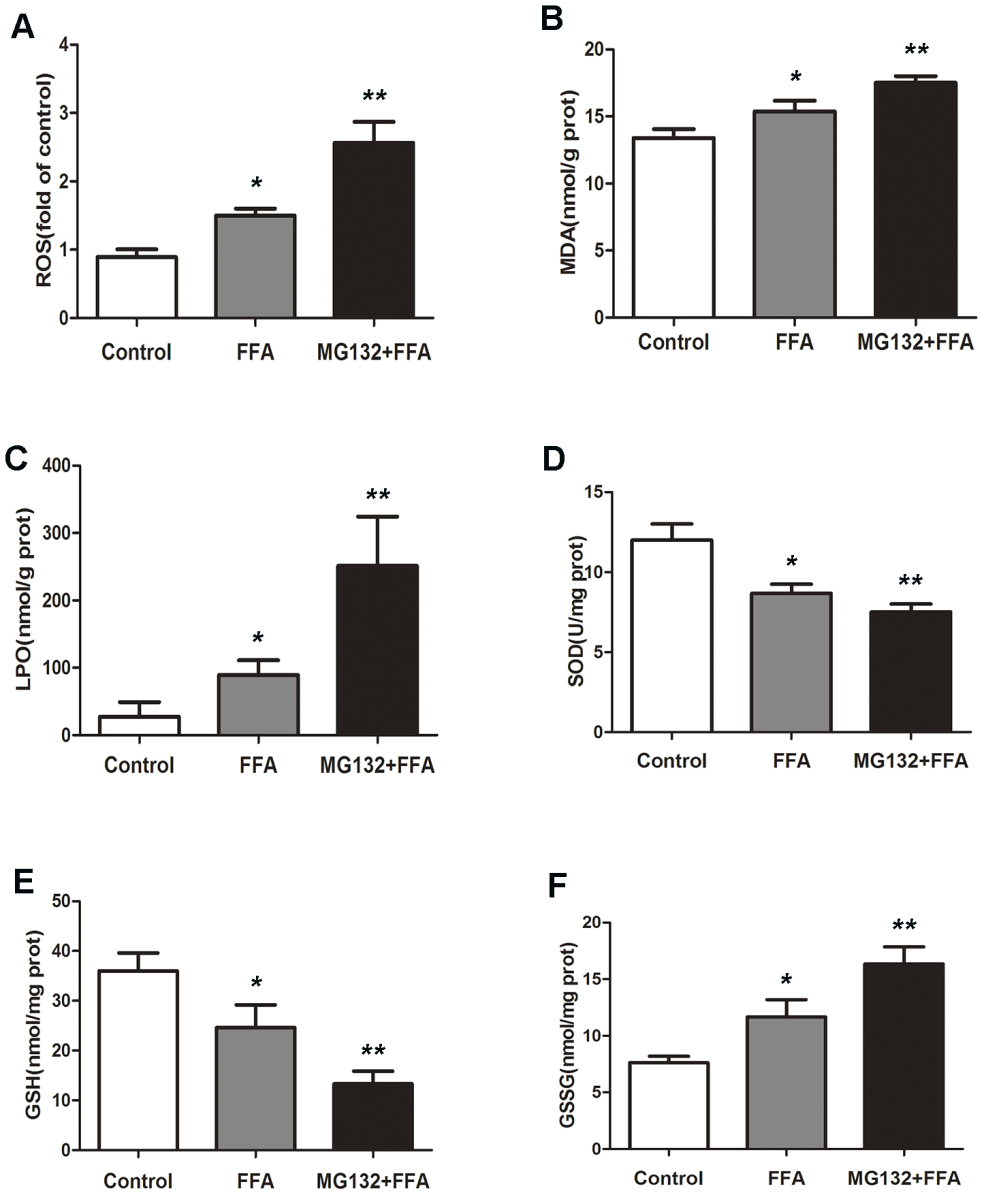
p21 overload aggravates oxidative stress accumulation in FFA-buffered L02 cells. L02 cells were treated with DMEM, 1 µM MG132, respectively for 48 hr. ROS (A), MDA (B), LPO (C), SOD (D), GSH (E) and GSSG (F) levels were measured using enzymatic kits. *p<0.05 vs DMEM treated L02 cells. **p<0.05 vs FFA treated L02 cells. ROS, reactive oxygen species; MDA, malondialdehyde; LPO, lipid peroxide; SOD, superoxide dismutase; GSH, glutathione; GSSG, oxidized glutathione.

To dissect the mechanisms underlying p21-mediated lipotoxic action in L02 cells, oxidative damage was assayed by measuring ROS, MDA, LPO, SOD, GSH and GSSG level. ROS, MDA, LPO and GSSG measurements showed that the level were increased by FFA treatment and were raised much more when added with MG132 (Figure 3A, 3B, 3C, 3F). Accordingly, the significant decrease of SOD and GSH level were correlated with enhanced LPO and GSSG level (Figure 3D, 3E). These observations implied that p21 overload exacerbated the oxidative accumulation in a dose-dependent manner, and exerted a significant synergist effect in L02 cells buffered with FFA.

### E. Silencing p21 expression alleviates FFA-induced steatosis in L02 cell

**Figure 4:**
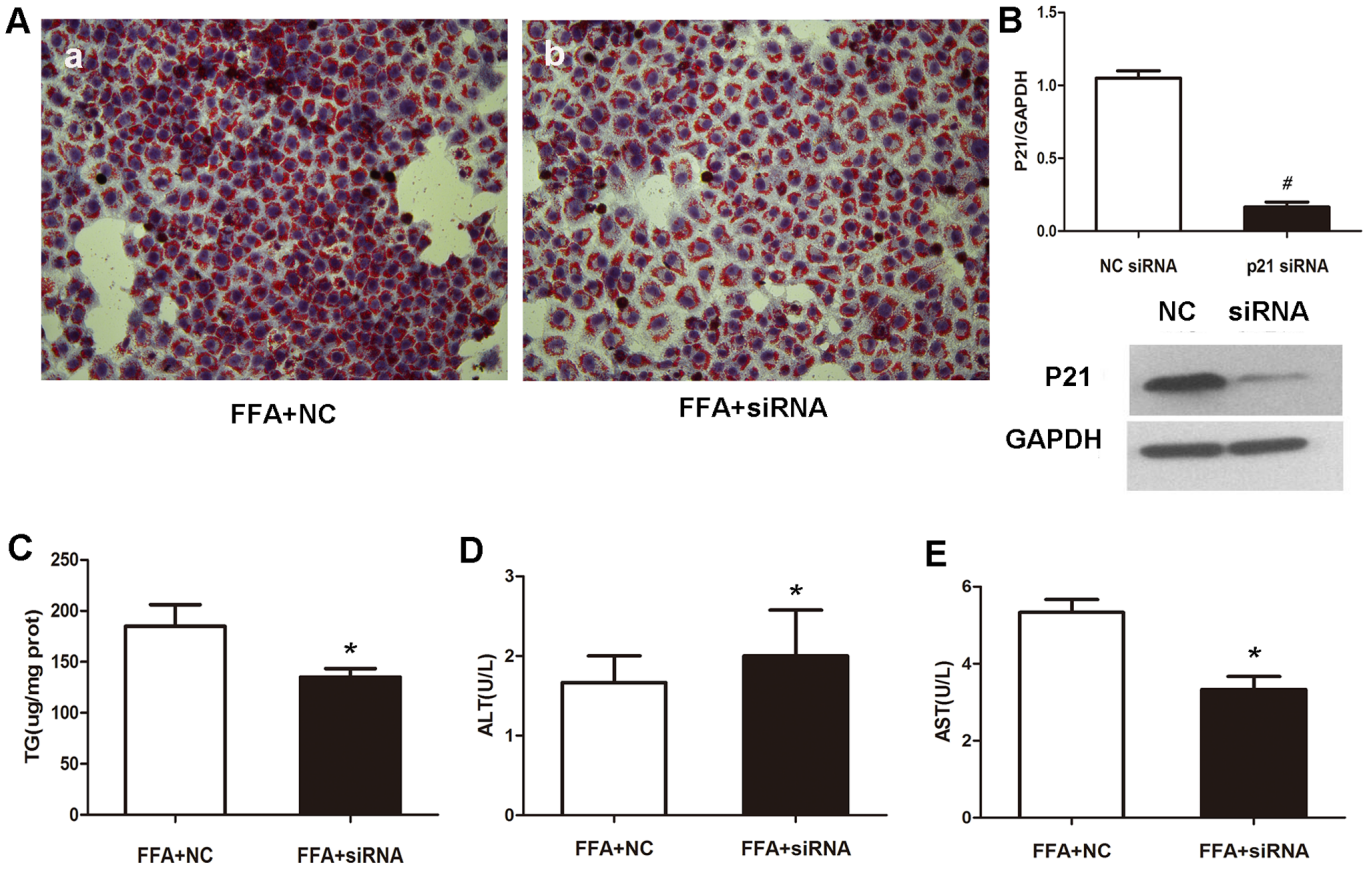
p21 knockdown alleviates FFA-induced steatosis in L02 cell. (A) Representative micrographs showing intracellular lipid accumulation in L02 cells as stained by Oil Red O (magnification, 200×). Panels a, b are L02 cells transfected with negative control siRNA, p21 siRNA respectively for 12 hr, then incubated in 1 mM FFA for 48 hr. (B) Western blot analysis of cellular p21 protein level in negative control or p21 siRNA treated group. Relative protein levels were used to quantify knockdown efficient compared to negative control. Values were means ± standard deviation. ^#^ *p*<0.01. After negative control or p21 siRNA treatment, L02 cells were incubated with 1 mM FFA for 48 hr. Cellular TG content (C), ALT level (D), AST level (E) were measured using enzymatic kits. Results were expressed as means ± standard deviation. *p<0.05 vs negative control group. NC, negative control; siRNA, p21 siRNA.

We then used a pairs of siRNA duplexes to down-regulate p21 expression in L02 cell lines. Western blot analysis confirmed marked drop of p21 protein level by significant inhibitory efficiency of siRNA compared to the negative control (Figure 4B). To investigate whether knockdown of p21 has a crucial role on hepatocyte lipid accumulation, L02 cells transfected with p21 siRNA and negative control siRNA were cultured in FFA mixtures.

As depicted in Figure 4A, Oil Red O staining demonstrated that lipid stores were present in negative control group when treated with FFAs for 48 hr, but markedly decreased in cells transfected with p21 siRNA. As expected, the siRNA knockdown of p21 resulted in a significant of 25% decrease in cellular TG content (Figure 4C). AST level significantly decreased but not AST with siRNA intervene (Figure 4D, 4E).

**Figure 5:**
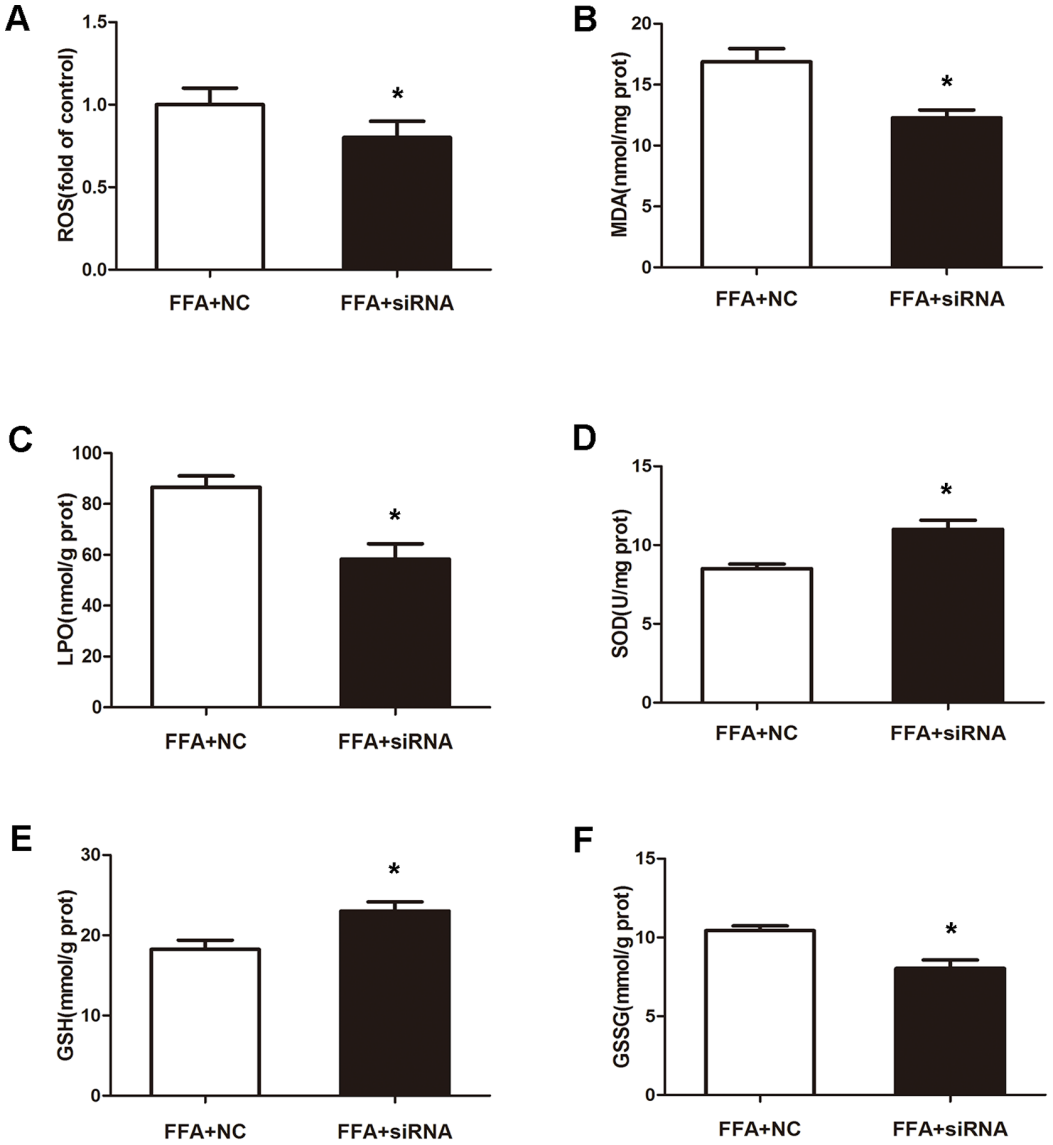
p21 knockdown alleviates FFA-induced oxidative stress in L02 cells. L02 cells were transfected with negative control or p21 siRNA, then incubated with 1(A), MDA (B), LPO (C), SOD (D), GSH (E) and GSSG (F) levels were measured using enzymatic kits. Values were means ± standard deviation. *p<0.05 vs negative control.

The extent of oxidative stress in the p21 knockdown model of cell steatosis was compared to that negative control. As seen in Figure 5, a sharp drop in ROS, MDA, LPO and GSSG levels was reached after FFA exposure (p<0.05). This was confirmed by the significant increase of SOD and GSH levels (Figure 5D, 5E). These results suggested that oxidative stress in FFA-induced cellular steatosis was alleviated by silencing p21 expression.

## Discussion

Understanding the pathogenesis of NAFLD has recently assumed great importance with the recognition that it has a high prevalence and has the potential to progress to fibrosis and cirrhosis.

NAFLD patients exhibit an elevated lipolysis and high circulating FFA levels [9]. High circulating FFA concentration may aggravate hepatic fat accumulation by disrupting lipid metabolism in NAFLD patients, and thus studying how FFA influences metabolic regulator will further improve our understanding about the pathogenesis of NAFLD [30].

In the process of FFA oxidation, the mitochondria leaks ROS mainly in the form of hydrogen peroxide [5]. Once the balance between intrahepatic antioxidants and ROS is broken, ROS triggers steatohepatits by lipid peroxidation, cytokine induction, and Fas ligand induction [5]. These findings suggest that FFA-overloading is not only a mediator of metabolic syndrome but also initiates lipotoxicity that accompanied by a range of histological alterations varying from simple steatosis to NASH, with time progression to manifest cirrhosis.

Rather than being an ‘innocent bystander’, recent studies suggest that oxidative stress-induced lipid peroxidation may be a guilty party playing important roles in the progression of NAFLD. Furthermore, it is confirmed that p21 expression correlates with oxidative stress in cell death [31], senescence [21] and hepatocyte regeneration [22], [23]. Long-term follow up clinical studies demonstrate higher liver p21 expression with disease progression in NAFLD [18], [19].

So it is reasonable to speculate a positive association between p21 and steatosis. The aim of the current study was to investigate the influence of p21 on NAFLD in a FFA-induced cell model, which may help elucidate the mechanism involved in the progression of the disease.

In our attempts to characterize the influence of p21 on NAFLD progression, we found this to be ROS-dependent and potentiate FFA-induced steatosis. The p21 level was higher after FFA treatment compared to DMEM buffered L02 cells. MG132 leads to p21 overload since the ubiquitin-dependent proteolysis of p21 is blocked. Compared to FFA treated LO2 cells, those with FFA plus MG132 intervene presented more severe cellular steatosis. And there was close association between cellular steatosis and oxidative stress induced lipid peroxidation. The oxidative stress level, identified by ROS, MDA, LPO and GSSG, together with the cytotoxic marker (AST), increased in parallel with intracellular lipid vacuoles (confirmed by Oil Red O staining) and TG content. Meanwhile, the anti-oxidant markers (SOD, GSH) decreased accordingly.

These findings suggest that FFA treatment in L02 cells causes lipid accumulation and oxidative stress, possibly mediated by p21, and it is a likely explanation for increased levels of p21 in NAFLD, reported previously.

Thus, we assumed that p21 may catalyze the first hit of oxidative stress. According to this hypothesis, a critically down-regulate expression of p21, alleviates oxidative stress and lipid peroxidation. Mirroring this, this study demonstrated a knockdown of p21 expression in L02 cells, with less lipid vacuoles, decreased TG content, improved cytotoxic injury (AST), elevated anti-oxidant level (SOD, GSH) and alleviated oxidative stress (ROS, MDA, LPO and GSSG) after 48 hr of FFA treatment compared to the negative control.

Our data are consistent with the hypothesis that increase of p21 in L02 cells causes oxidative stress and lipid peroxidation, and accelerates FFA-induced lipotoxic effect. And the p21 silencing L02 cells were less sensitive to FFA to develop steatosis.

The strong association of hepatocyte p21 expression with NASH, fibrosis stage and diabetes mellitus, has been studied a lot. Most of them attribute p21 as a hepatocyte senescence marker with an adverse liver-related outcome by its cell cycle arrest and apoptosis effect [20], [32], [33]. However, none of these studies looked at cellular p21 expression, oxidative stress or lipid peroxidation in relation to NAFLD. In contrast to these studies, we focused on the ‘first hit’ effect of p21 in NAFLD program. Our results provide evidence that the elevation of intracellular ROS levels is an important part of the mechanism by which p21 induces steatosis, and that is likely due to their effects on lipid peroxidation. These results emphasized the power of p21 in aggravating NAFLD progression and indicated that p21 up-regulation may contribute to the defect of mitochondrial fatty acid β-oxidation, which subsequently leads to TG storage in the liver. It is noteworthy that p21 may not only induce cell apoptosis or senescence, but also cause steatotic changes in hepatocytes that exacerbate NAFLD progression.

## Conclusions

In conclusion, this study demonstrated a link between p21 expression, oxidative stress and lipid peroxidation in NAFLD and further, p21 played lipotoxic effect on FFA-induced steatosis in L02 cells. Analysis of p21 effect provided both global and specific information regarding the molecular events that cause this complex disease. The strong relation between cellular p21 expression and impaired fatty acid oxidation may be a potential target for therapeutic intervention. However, the precise mechanism by which p21 promotes hepatic steatosis remains to be elucidated, and further investigations are underway.
